# Evaluation of Adipokines: Apelin, Visfatin, and Resistin in Children with Atopic Dermatitis

**DOI:** 10.1155/2013/760691

**Published:** 2013-02-14

**Authors:** Edyta Machura, Maria Szczepanska, Katarzyna Ziora, Dariusz Ziora, Elzbieta Swietochowska, Małgorzata Barc-Czarnecka, Alicja Kasperska-Zajac

**Affiliations:** ^1^Department of Pediatrics, Medical University of Silesia, Ulica 3-go Maja 13-15, 41-800 Zabrze, Poland; ^2^Department of Pneumonology and Tuberculosis, Medical University of Silesia, Ulica Koziołka 1, 41-803 Zabrze, Poland; ^3^Department of Biochemistry, Medical University of Silesia, Ulica Jordana 19, 41-808 Zabrze, Poland; ^4^Department of Internal Diseases, Allergology and Clinical Immunology, Medical University of Silesia, Ulica Ceglana 35, 40-952 Katowice, Poland

## Abstract

Very little is known about the role of adipokines in atopic dermatitis (AD) in children. This study aimed at analyzing the serum levels of resistin, apelin, and visfatin in children with AD in relation to body weight, AD severity, and gender. Serum concentration of adipokines was measured in 27 children with AD and in 46 healthy subjects. Selected biochemical parameters were evaluated and skin prick test was performed. Serum levels of resistin and apelin were significantly higher, whereas serum visfatin concentration was significantly lower in children with AD versus healthy controls, although an increase in resistin levels was exclusively demonstrated in boys. In AD group, a significant increase in apelin levels in girls was documented. There was no relationship between adipokines levels and the degree of allergic sensitization. Receiver operating characteristic curve analysis demonstrated that the serum apelin cutoff value differentiating children with AD from those without was >137.8 pg/mL. Resistin and visfatin cutoff values were >3.8 ng/mL and ≤ 2.13 ng/mL, respectively. Apelin and visfatin can serve as excellent indicators to distinguish children with AD from those without disease.

## 1. Introduction

 Increasing literature evidence indicates that obesity is a risk factor for the development of asthma [1, 2]. Exact mechanisms for the links between obesity and asthma are not well understood, although a possible role for adipokines has been indicated [3, 4]. 

Recent studies showed that adipose tissue is far more than a site for energy storage and it is in fact an active endocrine, paracrine, and also immune organ secreting multiple bioactive mediators, called adipokines. These adipokines include hormones (leptin, adiponectin), cytokines (TNF-*α*, IL-6, IL-10, and visfatin), and other proteins (apelin, resistin), which participate in numerous physiological and pathological processes [5, 6]. The deregulated synthesis and/or secretion of adipokines towards the proinflammatory compounds was observed in obesity and obesity-related disorders [5]. In addition, several studies suggest that some of adipokines are involved in the pathogenesis of other inflammatory disorders including asthma [7], inflammatory bowel disease [8], rheumatoid arthritis [9], and psoriasis [10]. Several reports pointed at the proinflammatory role of leptin and anti-inflammatory role of adiponectin in asthma, but the data are currently equivocal [4, 7]. It seems that another adipokine, resistin, may be also involved in asthma pathogenesis and severity [11]. Our recent studies revealed altered apelin and visfatin levels in childhood atopic asthma [12, 13].

In contrast to asthma, studies in children that investigate the relationship between obesity and AD are few but with controversial results [2, 14]. Also data related to the role and potential significance of adipokines in AD are limited [7]. Since AD is characterized as a chronic inflammatory disorder, we hypothesized that abnormalities in adipokine serum levels might occur. Therefore in the present study, we estimated apelin, visfatin, and resistin serum levels in children with AD. The adipokine concentrations were related to body weight and AD severity. Additionally, possible differences in adipokine levels between boys and girls were also explored.

## 2. Materials and Methods

Twenty-seven children (mean age 9.9 ± 0.77 range 4.3–17.5 y) suffering from mild to severe AD were enrolled into the study. AD was diagnosed according to the criteria described by Hanifin and Rajka [15]. The severity of dermatitis was estimated according to the SCORAD index (ranging from 0 to 103 points) [16]. Based on the total score, 6 children were classified as mild (<25 points), 12 children as moderate (25–50 points), and 9 children as severe (>50 points) AD. All the children had positive skin prick tests (SPTs) to ≥1 allergens. A positive SPT was defined as a mean diameter of at least 3 mm in the presence of negative diluents and positive histamine controls. The degree of allergic sensitization was measured by the whole size of SPTs. Children with concomitant asthma and rhinitis were excluded from this study. The patients were not treated with any antihistamines, topical steroids, or calcineurin inhibitors for at least 1 week before enrolment into the study (only emollients were applied). They were free of any systemic steroids during the preceding 8 weeks. The control group consisted of 46 healthy children (mean age 11.04 ± 0.62, range 4−17 y) with a negative history of allergic disease, the normal level of total serum IgE, and negative results of skin prick test to a panel of aeroallergens (dust mite, mixed grass or tree pollen, cat and dog; Allergopharma, Reinbek, Germany). Children included into the control group attended the outpatient pediatric clinic for nonimmunological, noninflammatory health problems and needed venous puncture.

The present study was approved by the Ethics Committee of the Medical University of Silesia in Katowice. Written informed consent was obtained from children's parents.

### 2.1. Specific Measurements 

#### 2.1.1. Anthropometric Measurements

Body mass index (BMI) (body weight (kg)/height squared (m^−2^), and standard deviation (SD) score for BMI (BMI-SDS) were calculated according to the current Polish population normal range [17].

#### 2.1.2. Biochemical Measurements

The fasting blood samples were collected in the morning between 7:00 AM and 9:30 AM following an overnight fasting. The serum was separated by centrifugation at 1300 g for 10 min at +4°C and immediately stored at −70°C until analyzed. Biochemical parameters, including C-reactive protein (CRP), triglycerides (TGs), total cholesterol (TC), and high-density lipoprotein cholesterol (HDL-C), were measured, using chemistry analyzer (Cobas 6000, Roche Diagnostic, Switzerland). Low-density lipoprotein cholesterol (LDL-C) was calculated by the Friedewald formula (LDL-C = TC − HDL-C − TG/5).

#### 2.1.3. Apelin, Visfatin, and Resistin Measurements

Serum visfatin C-terminal and apelin-12 concentrations were measured by commercial enzyme immunoassay kit according to the manufacturer's protocol (human recombinant visfatin C-terminal, human recombinant apelin-12 (Phoenix Pharmaceuticals, Inc., Burlingame, CA, USA)). The concentrations of apelin-12 and visfatin C-terminal were determined on the basis of standard curve carried out for serial dilution available in kit standards. Absorbance was measured using spectrophotometer (*μ*Quant, Microplate Reader, Bio-Tek, Winooski, VT, USA) at 450 nm wavelengths. Acquired data were analyzed using KC Junior Software (v.1.31.5, Bio-Tek Instruments, Winooski, VT, USA). The sensitivity values for visfatin and apelin-12 were 2.17 ng/mL and 0.07 ng/mL, respectively. The intra- and interassay errors for visfatin were <10% and <15% and 8.2% and <15% for apelin-12, respectively. 

Serum resistin concentration was measured by commercially available enzyme-linked immunosorbent assay kit (Mediagnost, Reutlingen, Germany). The concentration of resistin was determined on the basis of standard curve carried out for serial dilution available in kit standards (human recombinant resistin). Immunocomplex detection was determined on the basis of reaction with rabbit polyclonal antibody antihuman immunoglobulin (IgG) conjugated with horseradish peroxides and then with TMB substrate solution (TMB substrate, slow kinetic, Sigma-Aldrich, St. Louis, MO, USA). Absorbance was measured using spectrophotometer (*μ*Quant, Microplate Reader, Bio-Tek, Winooski, VT, USA) at 450 nm wavelengths. Acquired data were analyzed using KC Junior Software (v.1.31.5, Bio-Tek Instruments, Winooski, VT, USA). The sensitivity for resistin was 0.012 ng/mL, and the intra- and interassay errors were 4.66% and 4.79%, respectively. 

### 2.2. Statistical Analysis

The statistical analysis was performed using Statistica 6.0 software (StatSoft, Inc., Tulsa, OK, USA) and data presented as mean values ± SE. Mann-Whitney *U* test was used for comparisons between groups. Correlations were calculated using the Spearman rank test. Accuracy of the diagnostic adipokines measurement test was assessed using the receiver operating characteristic (ROC) curve analysis. ROC analysis was performed using MedCalc software (v11.3.5.0). For all tests, values of *P* < 0.05 were considered statistically significant.

## 3. Results

Characteristics of the 27 children with AD and the 46 healthy control subjects are presented in Table 1. Both groups were similar in age (*P* = NS). In AD group, 25.79% (*n* = 6) of children were obese as well as 13.04% (*n* = 6) in healthy children. Despite this, the mean values of BMI and BMI-SDS of AD children were similar as compared to those of the healthy group. Normal weight was defined as BMI-SDS between −2.0 and +2.0. Obesity was defined as BMI-SDS > 2.0. Underweight was defined as BMI-SDS <−2. Children with AD had higher IgE levels and higher percentage and blood eosinophil numbers.

### 3.1. Comparison of Serum Adipokine Levels between Children with AD and Control Group

Mean values of serum levels of apelin, visfatin, and resistin in all children are shown in Table 2. Serum levels of apelin and resistin were significantly higher in the total group of AD children than those of control group (*P* < 0.001, *P* < 0.003). However, the increase in the resistin level was seen only in boys. In contrast, serum visfatin level was significantly lower in AD children as compared to that of healthy subjects (*P* < 0.001). In AD children, after stratifying by gender, there was a significant increase in apelin levels in girls as compared to those of boys (*P* < 0.01). On the other hand, no differences in any measured clinical and laboratory parameters, including age, BMI, BMI-SDS, or severity of disease between boys and girls, were observed. In the control group, serum levels of all adipokines were significantly higher in girls than in boys (*P* < 0.001).

### 3.2. Comparison of Serum Adipokine Levels/BMI Ratio between Children with AD and Control Group

As serum adipokine levels are dependent on the amount of adipose tissue, adipokine levels were adjusted for BMI by dividing the measured concentration by BMI. Mean values of adipokine serum levels/BMI ratios are shown in Table 3. We found that apelin levels/BMI ratio was significantly higher and visfatin level/BMI ratio was lower in children with AD when compared with healthy children (*P* < 0.001). However, a significant increase in apelin level/BMI ratio was seen only in girls. The apelin/BMI ratio showed no difference in comparison to the control group (*P* = 0.08). No difference between BMI adjusted serum concentrations of resistin between AD children and healthy subjects was found. After stratifying by gender, there was no difference in adipokines/BMI ratio in the particular groups both in AD and in healthy children. Among the 73 children enrolled in this study, there were 18 normal-weight children with AD and 38 healthy normal weights. No differences in serum resistin, visfatin, and apelin levels between normal weight and obese AD children were observed as well (data not shown).

### 3.3. Correlations between Adipokines and Clinical and Laboratory Parameters

We have documented only few significant correlations between adipokines levels and other parameters (Table 4).

In children with AD, a strong positive correlation was found between visfatin concentration and TG levels. In addition, serum apelin levels correlated positively with serum visfatin levels. Serum resistin levels inversely correlated with age of AD children. Adipokines showed no correlation with BMI and BMI-SDS, SCORADindex, duration of AD, and with degree of allergic sensitization. SCORADindex values correlated exclusively with serum IgE levels. No correlation was found between BMI and severity of AD.

No correlation between age, BMI and BMI-SDS, IgE, and serum adipokines was found in examined healthy children, but strong positive correlation between serum TG levels and resistin, visfatin, and apelin was observed. In addition in control group, serum levels of resistin correlated significantly with both serum visfatin and apelin levels; also serum apelin level correlated with serum visfatin level.

**Figure 1:**
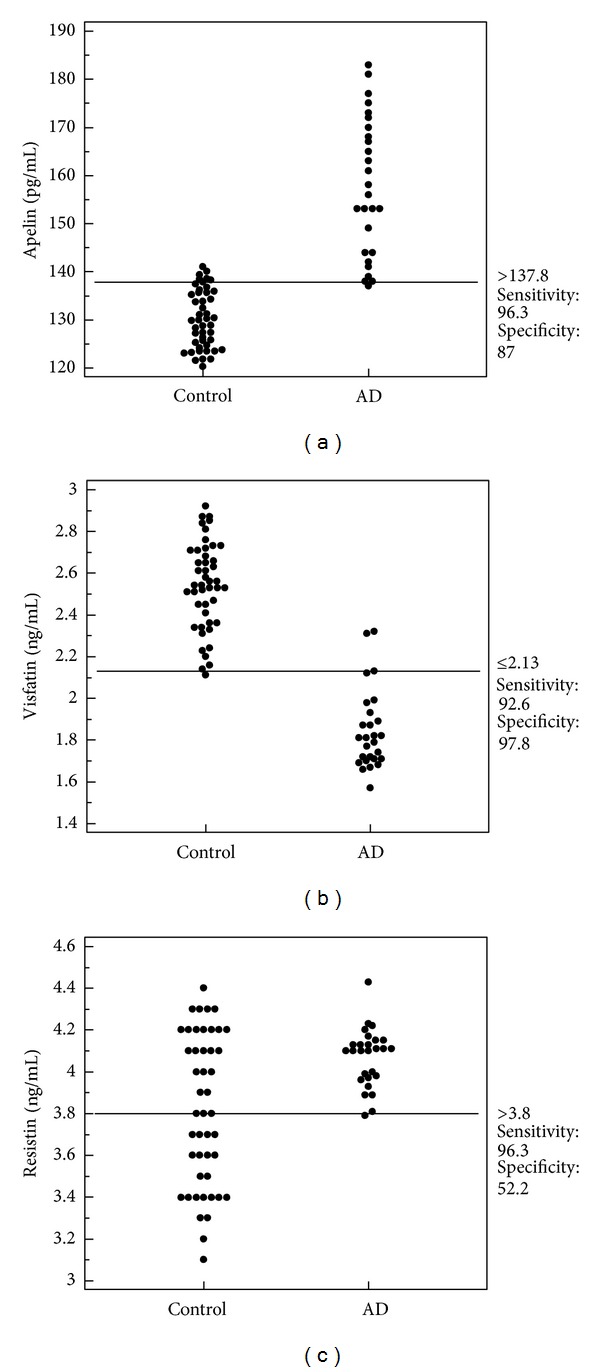
Interactive dot diagram comparing levels of (a) apelin, (b) visfatin, and (c) resistin levels between children with atopic dermatitis and healthy children.

**Figure 2:**
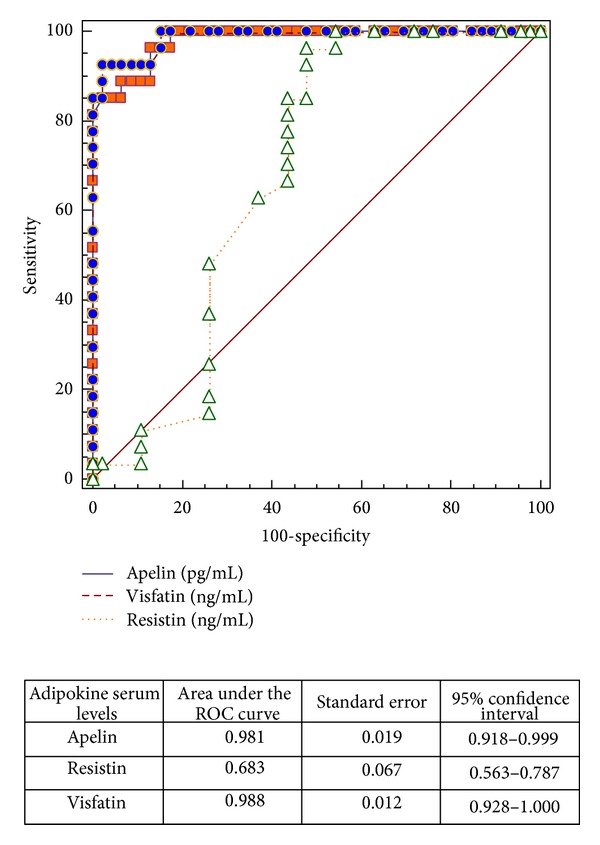
Comparison of receiver operating characteristic (ROC) curves for apelin, resistin, and visfatin between children with atopic dermatitis and healthy children.

In order to evaluate the diagnostic value of serum adipokine levels for differentiation children with AD from all examined children, we performed receiver operating characteristic analysis (ROC). When the cutoff value for apelin was set to 137.8 pg/mL, the sensitivity and specificity for AD were 96.3% (95% CI: 81.0–99.9) and 86.96% (95% CI: 73.7–95.1), respectively. For resistin, the cut-off value was ≥3.8 ng/mL, the sensitivity and specificity for AD were 96.3% (95% CI: 81.0−99.0) and 52.17 (95% CI: 36.9–67.1), and for visfatin the cut-off value was ≤2.13, the sensitivity and specificity were 92.59% (95% CI: 75.7–99.1%) and 97.83 (95% CI: 88.5–99.9%) (Figure 1). The area under the ROC for apelin was 0.981, and for visfatin was 0.988 indicating that serum levels of both adipokines are the best biomarkers differentiating subjects with atopic dermatitis from healthy children (Figure 2).

## 4. Discussion

The current study was designed to examine the circulating apelin, resistin, and visfatin levels in children with AD, who had no current additional allergic symptoms such as asthma and allergic rhinitis. To eliminate the possible effect of obesity on adipokine levels, BMI adjusted serum concentration of adipokines was also assessed. We demonstrated that in children with AD elevated apelin concentrations and apelin/BMI ratio are accompanied by decreased visfatin levels and visfatin/BMI ratio. However, an increase in apelin/BMI ratio was seen only in girls. Moreover, the difference in all adipokine levels between AD and healthy children was found, despite similar body weight. This finding suggests that these adipokines may be implicated in the immunopathogenesis of AD, independently of their confirmed role in obesity.

Data about the behaviour of adipokines in AD are limited. Only three studies evaluating leptin or adiponectin in AD were conducted. Nagel et al. showed a negative association between serum adiponectin levels and atopic dermatitis, and ever diagnosed eczema, and found no association between circulating leptin concentration and AD symptoms in children [7]. They also did not confirm the significant difference in serum leptin between AD children and control group. It was in accordance with the study of Bostanci et al. This manuscript has documented additionally that local steroid application did not influence circulating leptin levels [18]. In contrast to this data, Kimata documented elevated serum leptin in AD children [19]. The role of adipokines such as apelin, visfatin, and resistin in patients with AD has not been investigated so far.

Apelin is a more recently identified adipokine, which exists in different isoforms, and acts thorough the binding to a specific G-protein-coupled receptor named API, present on endothelial cells, vascular smooth myocytes, and cardiomyocytes [20, 21]. Apelin synthesis in adipocytes is strongly upregulated by insulin, and plasma apelin level markedly increases in obese and hyperinsulinemic mice and humans [22] its excretion is induced by hypoxia [23]. In addition, TNF-alpha may act as a key player in the upregulation of apelin expression in adipocytes both in obese and lean human [24]. It has been reported that apelin has a regulatory effect on cardiovascular system, appetite, drinking behaviors, neoangiogenesis, lymphangiogenesis, fibrogenesis, and tumor growth [25, 26]. However, the data on apelin are controversial. Lately, Reinehr et al. in their longitudinal study revealed the lack of association between apelin and obesity in children [27]. 

The possible role of apelin in allergic inflammation has not been widely studied in both human and animal models, yet. However, in our previous papers we confirmed high apelin concentration in children with asthma [12]. 

The origin and the role of increased apelin levels in serum among examined children with AD remain unclear. Since neoangiogenesis is a feature of AD skin lesion [28], it seems that apelin may be involved also in this chronic inflammatory disorder.

 Surprisingly, among children with AD we observed a decreased serum level of visfatin compared with healthy children, and this finding suggests that visfatin may have a protective effect against AD in children. We have also documented in children with atopic asthma similar reduced concentration of visfatin [13]. In this study, we excluded children presenting simultaneously with both AD and asthma symptoms to link the adipokines changes exclusively to AD. Visfatin **(**PBEF-pre-B cell, colony-enhancing factor) is a growth factor for early B-lymphocytes, and it is highly expressed and secreted by human visceral fat (hence the term “visfatin”) [29]. Plasma concentration of visfatin strongly correlates with the amount of fat. Visfatin upregulates the production of the pro- and anti-inflammatory cytokines, for example, interleukin-1*β*, (IL-1*β*), TNF-*α*, IL-1Ra, IL-6, and IL-10 and increases the surface expression of costimulatory molecules, for example, CD54, CD40, and CD80 in monocytes [8]. Moreover, visfatin enhances production of chemokines such as CXCL8, CXCL10, and CCL20 in human keratinocytes [30]. Increased visfatin expression and/or visfatin plasma concentrations have been identified in a variety of chronic inflammatory diseases, including rheumatoid arthritis [9], inflammatory bowel disease [8], and psoriasis [10]. There are conflicting reports about the relationship between obesity and circulating levels of visfatin. Some researchers reported increased visfatin plasma levels in obese adult women [31] or obese children [32], whereas others observed no apparent relationship between overweight and/or obesity and serum visfatin concentrations [33], or even noted a negative correlation [34].

 We observed significantly higher levels of serum resistin in AD than in controls, but this feature was seen only in boys. However, BMI adjusted serum concentration of resistin was similar in both groups. Our results suggest that the global increase in resistin levels in our AD boys might correlate with the amount of adipose tissue. Further studies should explore if resistin may be one of the factors explaining the relation between obesity and AD. There are only few publications on resistin in human asthma. One study of children showed a protective association between serum resistin concentration and risk for asthma [35]. On the contrary, LaRochelle et al. documented that plasma resistin levels were significantly higher in adult patients with moderate-to-severe persistent asthma than in control and increased with disease severity [11]. However, Arshi et al. did not find any difference in resistin levels between children with asthma and healthy children [36]. Resistin is expressed in brown and white adipose tissues and is a member of the family of cysteine-rich proteins called resistin-like molecules [37]. Circulating monocytes and macrophages seem to be responsible for resistin production in human [5]. Resistin has proinflammatory properties and upregulates the expression of monocyte chemoattractant protein 1 (MCP-1) as well as vascular cell adhesion molecule 1 (VCAM-1) and intercellular adhesion molecule 1 (ICAM-1) in endothelial cells, which have been known to contribute in the pathogenesis of allergic inflammation [5]. Macrophage exposure to resistin has been shown to induce expression of tumor necrosis factor-alpha (TNF-alpha) and IL-12 and IL-6 [38]. 

 In the current study, also obese AD children had similar levels of all adipokines as normal-weight AD children. The lack of relationship between adipokine levels and BMI or BMI-SDS may be explained by differences in fat distribution. Unfortunately in this study, the measures of central obesity were not estimated. Since visceral fat is supposed to be more metabolically active than subcutaneous fat, the correlation of adipokine levels and central obesity requires further evaluation. We have not found difference in serum adipokine levels in different degrees of both AD severity and allergic sensitization and total IgE. Additionally, the degree of AD severity showed no correlation with obesity. Recently, Silverberg et al. in a retrospective case-control study found an association between prolonged obesity (>2,5 years) in early childhood and increased odds ratio of AD and severity of AD. Nevertheless, they performed their examination without the application of a widely established AD scoring systems (SCORAD, Eczema Area and Severity Index) [2]. 

 We demonstrated a significant difference in serum apelin levels between boys and girls including children with AD. In the control group, sex differences in apelin, resistin, and visfatin levels were observed. It should be emphasized, that other studies in children and adolescents have shown girls to have greater resistin concentration [39], but gender-related differences in visfatin levels were not observed [31]. The most interesting finding, which is the strength of the current study, is that apelin and visfatin serve as good markers differentiating AD children from healthy ones. However, it is difficult to establish if altered adipokine levels play a causative role in AD or if they have changed secondary to chronic allergic inflammation.

 The study has some limitations. Firstly, the sample size was rather small to represent all the children with atopic dermatitis. This was because the children were selected according to specific criteria—high total and specific IgE or positive SPT, without rhinitis or asthma. It is very difficult to collect an extensive number of AD children without concomitant disorder which may affect adipokine concentrations. Secondly, the wide range of age in examined children with AD, although similar to control, may be considered as a drawback of this study. Thirdly, a cross-sectional analysis of this study precludes any temporal or cause-effect conclusions. 

 In conclusion, this preliminary study provides the evidence of significantly higher apelin and decreased level of visfatin in atopic dermatitis. Additional prospective studies with a larger number of patients and application of other adipokines are needed to clarify the precise mechanism and their significance in the immunopathogenesis of AD in children. 

## Figures and Tables

**Table 1:** Demographic and clinical characteristics of children with atopic dermatitis and healthy children.

Characteristics	Children with AD (*n* = 27)	Healthy children (*n* = 46)
Age, years, mean ± SE	9.9 ± 0.99	11.7 ± 3.7
Sex, *n*	M: 10; F: 17	M: 19; F: 27
Duration of AD, years, mean ± SE	6.24 ± 0.37	—
SCORAD, mean ± SE	47.67 ± 67	

Serum total IgE, IU/mL, mean ± SE	768.23 ± 174.3^#^	50.11 ± 0.93
Eosinophils, % (*n*, cells/*μ*L), mean ± SE	8.5 ± 1.38^‡^	2.21 ± 0.4
	(488.4 ± 80.61)^†^	(155.05 ± 13.42)

Positive SPT, *n* (%)	100 (100)	0
Total SPT wheal size, mm, mean ± SE	22.9 ± 2.43	—
BMI	18.1 ± 1.44	18.76 ± 0.56
BMI-SDS	0.83 ± 0.41	0.18 ± 0.26
CRP (mg/L)	2.68 ± 1.9	1.01 ± 0.2
TG (mmol/L)	1.09 ± 0.13	0.97 ± 0.04
TC (mmol/L)	4.34 ± 0.015	3.99 ± 0.03
LDL-C (mmol/L)	2.29 ± 0.015	1.99 ± 0.06
HDL-C (mmol/L)	1.86 ± 0.01	1.5 ± 0.02

BMI: body mass index; BMI-SDS: body mass index-standard deviation scores; CRP: C-reactive protein; FEV_1_, HDL-C: high-density lipoprotein cholesterol; IgE: immunoglobulin E; IU: international unit; LDL-C: low-density lipoprotein cholesterol; SE: standard error; SPT: skin prick test; TC: total cholesterol; TG: triglyceride.

All *P* values from Mann-Whitney's *U*-test. ^‡^ *P* < 0.05, ^†^ *P* < 0.01, and ^#^ *P* < 0.0002.

**Table 2:** Mean values of adipokine serum levels in children with atopic dermatitis and healthy children.

Mean adipokine serum levels	Children with AD (*n* = 27; M/F: 10/17)	Healthy children (*n* = 46; M/F: 19/27)
Apelin, pg/mL		
All subjects	157.52 ± 2.76	130.18 ± 0.89*
Boys	149.10 ± 4.42	124.69 ± 0.76*
Girls	162.47 ± 3.01^‡^	134.05 ± 0.84^∗∧^
Visfatin, ng/mL		
All subjects	1.84 ± 0.04	2.54 ± 0.03*
Boys	1.78 ± 0.03	2.37 ± 0.03*
Girls	1.88 ± 0.05	2.66 ± 0.03^∗∧^
Resistin, ng/L		
All subjects	4.07 ± 0.03	3.82 ± 0.05^†^
Boys	4.04 ± 0.05	3.46 ± 0.04*
Girls	4.09 ± 0.03	4.07 ± 0.04^∧^

Data as shown as mean ± standard error. *P* values from Mann-Whitney's *U*-test. *P < 0.001 AD versus control.

^†^ *P* < 0.003 AD versus control.

^‡^ *P* < 0.01 AD girls versus AD boys.

^*∧*^ *P* < 0.001 healthy girls versus healthy boys.

**Table 3:** The adipokine serum levels/BMI ratio in AD children and healthy controls.

Adipokine serum levels/BMI	Children with AD (*n* = 27; M/F: 10/17)	Healthy children (*n* = 46; M/F: 19/27)
Apelin pg/mL/BMI (kg/m^2^)		
All subjects	9.06 ± 0.20	7.18 ± 0.19*
Boys	8.54 ± 0.28	7.3 ± 0.66
Girls	9.37 ± 0.42	7.09 ± 0.36*
Visfatin ng/mL BMI/(kg/m^2^)		
All subjects	0.11 ± 0.00	0.14 ± 0.00*
Boys	0.10 ± 0.01	0.14 ± 0.01*
Girls	0.11 ± 0.01	0.14 ± 0.01*
Resistin ng/mL/BMI (kg/m^2^)		
All subjects	0.23 ± 0.01	0.21 ± 0.01
Boys	0.23 ± 0.02	0.20 ± 0.01
Girls	0.24 ± 0.01	0.21 ± 0.01

Data as shown as mean ± standard error. *P* values from Mann-Whitney's *U*-test.*AD versus control *P* < 0.001.

**Table 4:** Significant correlations between adipokine serum levels and other parameters in children with atopic dermatitis and healthy children.

Examined significant correlation	*R*	*P*
Atopic dermatitis		
Visfatin and triglycerides	0.97	0.02
Visfatin and apelin	0.49	0.008
Resistin and age	−0.41	0.03
SCORAD and IgE	0.65	0.0002
Healthy controls		
Visfatin and TG	0.97	0.03
Resistin and TG	0.99	0.001
Apelin and TG	0.99	0.0003
Resistin and apelin	0.98	0.019
Resistin and apelin	0.9	0.004
Apelin and visfatin	0.96	0.03

Spearman's rank test, *P* < 0.05; TG: triglyceride.
